# Wide spectrum of NR5A1‐related phenotypes in 46,XY and 46,XX individuals

**DOI:** 10.1002/bdrc.21145

**Published:** 2016-12-29

**Authors:** Sorahia Domenice, Aline Zamboni Machado, Frederico Moraes Ferreira, Bruno Ferraz‐de‐Souza, Antonio Marcondes Lerario, Lin Lin, Mirian Yumie Nishi, Nathalia Lisboa Gomes, Thatiana Evelin da Silva, Rosana Barbosa Silva, Rafaela Vieira Correa, Luciana Ribeiro Montenegro, Amanda Narciso, Elaine Maria Frade Costa, John C Achermann, Berenice Bilharinho Mendonca

**Affiliations:** ^1^ Sorahia Domenice, Aline Zamboni Machado, Bruno Ferraz‐de‐Souza, Antonio Marcondes Lerario, Mirian Yumie Nishi, Nathalia Lisboa Gomes, Thatiana Evelin da Silva, Rosana Barbosa Silva, Luciana R. Montenegro, Amanda Narciso, Elaine Maria Frade Costa, and Berenice Bilharinho Mendonca are from the Laboratório de Hormônios e Genética Molecular (LIM/42)Unidade de Endocrinologia do Desenvolvimento, Disciplina de Endocrinologia e Metabologia do Hospital das Clínicas, Faculdade de Medicina, Universidade de São PauloSão PauloBrasil; ^2^ Frederico Moraes Ferreira is from the Ciências da SaúdeUniversidade Santo Amaro, São Paulo, Brasil and Laboratorio de Imunologia, Instituto do Coração, Faculdade de Medicina, Universidade de São PauloSão PauloBrasil; ^3^ Lin Lin and John C. Achermann are form the Genetics & Genomic MedicineUniversity College London (UCL) Great Ormond Street Institute of Child Health, University College LondonLondonUnited Kingdom; ^4^ Rafaela V. Correa is from the Núcleo de Atenção Médica Integrada (NAMI)Universidade de FortalezaCearáBrasil

**Keywords:** disorders of sex development, NR5A1 gene, gonadal dysgenesis, primary ovarian failure, ovotestis, adrenal insufficiency

## Abstract

Steroidogenic factor 1 (NR5A1, SF‐1, Ad4BP) is a transcriptional regulator of genes involved in adrenal and gonadal development and function. Mutations in *NR5A1* have been among the most frequently identified genetic causes of gonadal development disorders and are associated with a wide phenotypic spectrum. In 46,XY individuals, NR5A1‐related phenotypes may range from disorders of sex development (DSD) to oligo/azoospermia, and in 46,XX individuals, from 46,XX ovotesticular and testicular DSD to primary ovarian insufficiency (POI). The most common 46,XY phenotype is atypical or female external genitalia with clitoromegaly, palpable gonads, and absence of Müllerian derivatives. Notably, an undervirilized external genitalia is frequently seen at birth, while spontaneous virilization may occur later, at puberty. In 46,XX individuals, *NR5A1* mutations are a rare genetic cause of POI, manifesting as primary or secondary amenorrhea, infertility, hypoestrogenism, and elevated gonadotropin levels. Mothers and sisters of 46,XY DSD patients carrying heterozygous *NR5A1* mutations may develop POI, and therefore require appropriate counseling. Moreover, the recurrent heterozygous p.Arg92Trp NR5A1 mutation is associated with variable degrees of testis development in 46,XX patients. A clear genotype‐phenotype correlation is not seen in patients bearing *NR5A1* mutations, suggesting that genetic modifiers, such as pathogenic variants in other testis/ovarian‐determining genes, may contribute to the phenotypic expression. Here, we review the published literature on NR5A1‐related disease, and discuss our findings at a single tertiary center in Brazil, including ten novel *NR5A1* mutations identified in 46,XY DSD patients. The ever‐expanding phenotypic range associated with *NR5A1* variants in XY and XX individuals confirms its pivotal role in reproductive biology, and should alert clinicians to the possibility of NR5A1 defects in a variety of phenotypes presenting with gonadal dysfunction. Birth Defects Research (Part C) 108:309–320, 2016. © 2016 The Authors Birth Defects Research Part C: Embryo Today: Reviews Published by Wiley Periodicals, Inc.

## Introduction

*NR5A1* (nuclear receptor subfamily 5 group A, member 1), previously known as *SF1* (Steroidogenic Factor‐1) and *Ad4BP* (Adrenal 4‐Binding Protein), was first cloned by Parker and colleagues in 1992, in an attempt to identify a protein that could activate the promoters of steroid hydroxylase enzymes (Lala et al., 1992).

*NR5A1* is expressed in steroidogenic tissues (Honda et al., 1993; Ikeda et al., 1993; Morohashi et al., 1994; Parker and Schimmer, 1997; Ramayya et al., 1997; Morohashi, 1999), pituitary gonadotrophs (Barnhart and Mellon, 1994; Ingraham et al., 1994; Ngan et al., 1999), and neurons located at the dorsomedial portion of the ventromedial hypothalamus (VMH) (Ramayya et al., 1997; Morohashi et al., 1999). In developing embryos, *Nr5a1* is expressed in the urogenital ridge, representing the first marker of gonadal and adrenal differentiation (Ikeda et al., 1994; Morohashi et al., 1995, 1999; Ramayya et al., 1997; Hanley et al., 1999, 2001). Throughout human development, NR5A1 is expressed in the steroid‐secreting adrenal cortex, in Leydig and Sertoli cells, as well as in granulosa and theca cells.

*NR5A1* stimulates the expression of several genes required for the development and maintenance of the male differentiation cascade. It regulates the expression of *LHCGR* and the steroidogenic enzymes *STAR, CYP11A1*, and *CYP17A1* in Leydig cells, required for testosterone biosynthesis. NR5A1 also increases the expression of insulin‐like polypeptide 3 (INSL3), which regulates testicular descent and is a survival factor for male germ cells in adults (Zimmermann et al., 1998; Tremblay and Robert, 2005). Anti‐Mullerian hormone (AMH) and its receptor, AMHR2, essential factors for male reproductive tract development, are also regulated by NR5A1. In Sertoli cells, NR5A1 regulates the expression of the testis‐determining genes *SRY* and *SOX9* (Jeyasuria et al., 2004).

Mutations in *NR5A1* are emerging as a frequent genetic cause of human 46,XY disorders of sex development (DSD), having been identified throughout the globe. In South America, familial and sporadic DSD patients bearing NR5A1 defects have been described in Brazil and Argentina (Lourenço et al., 2009; Ciaccio et al., 2012; Gabriel Ribeiro de Andrade et al., 2014; Fabbri et al., 2016). Here, we review the phenotype associated with ten novel and one previously described *NR5A1* allelic variants identified in a Brazilian cohort of 46,XY and 46,XX DSD patients followed at a single tertiary center. Our findings are discussed in light of previously published reports, and several aspects of the phenotypic spectrum associated with NR5A1 mutations in humans are reviewed.

## Patients

This is a retrospective study approved by the Ethics Committee of Hospital das Clinicas, University of Sao Paulo Medical School. Written informed consents were obtained from all patients or their parents/guardians.

The full cohort of DSD patients followed at the Developmental Endocrinology Unit at Hospital das Clinicas consisted of 83 patients with 46,XY DSD (48 patients with 46,XY gonadal dysgenesis and 35 patients with 46,XY DSD of unknown cause) and 90 patients with 46,XX DSD (70 patients with primary ovarian failure (44 patients had primary amenorrhea and 26 patients had secondary amenorrhea) and 20 patients with 46,XX SRY negative disorders of ovary development (16 with 46,XX ovotesticular DSD (OTDSD) and 4 with 46,XX testicular DSD (TDSD)).

## Molecular Analysis

Mutational analysis of *NR5A1* (Ensembl transcript ESNT00000373588) was performed by Sanger sequencing and by targeted massively parallel sequencing (TMPS), as detailed in Supporting Information.

Identified allelic variants were analyzed according to the joint American College of Medical Genetics and Genomics Association for Molecular Pathology Guidelines (Richards et al., 2015) (see Supporting Information)

### In Silico Analysis

Alignment of wild‐type and mutated *NR5A1* sequences was performed using MUSCLE (Edgar, 2004) and the NCBI's Reference Sequence (RefSeq) database. To evaluate the impact of NR5A1 nonsynonymous mutations at the tertiary protein structure, several structural models were built for wild‐type and mutated molecules in the YASARA suite (Krieger et al., 2004), using as templates the crystal structures of the human liver receptor homologue 1 (LRH‐1, NR5A2) DNA‐binding domain (DBD) in complex with the *hCYP7A1* promoter (PDB accession code 2A66), and the human NR5A1 ligand‐binding domain (LBD) in complex with di‐pamitoyl‐3‐SN‐phosphatidylethanolamine (PDB accession code 1ZDT). Stereo chemical quality of generated structural models was accessed with MOLPROBITY (Davis et al., 2007). Two molecular mechanics simulations were carried in explicit solvent for each model. Trajectories were calculated with the YAMBER3 force field. Ten thousand simulation steps of 2.5 fs were recorded for each wild type and its corresponding mutated molecules. The final confirmation of the lowest energy models was confirmed for each pair of simulations. The volumes of the cavities of the models were calculated using KVFINDER (Oliveira et al., 2014). Molecular models were drawn using PyMOL (www.pymol.org).

### In Vitro Functional Studies

Transient gene expression assays were performed using human embryonic kidney TSA‐201 cells to analyze transcriptional activation of murine *Cyp11a1* and rat *Cyp19* promoters by wild‐type or mutant NR5A1 (Lin et al., 2007) (see Supporting Information).

## Results

We identified 11 pathogenic *NR5A1* variants, including 10 novel variants in 46,XY DSD patients and one previously reported variant in a 46,XX testicular DSD patient.

### Clinical and Hormonal Characteristics of 46,XY DSD Patients Bearing Pathogenic *NR5A1* Variants

Nine of ten 46,XY DSD patients presented with atypical genitalia at birth and one had female external genitalia. Nine patients were assigned as female at birth, one of whom reassigned to male at 9 years of age (patient 9, Table 1); only one patient was assigned as male at birth (patient 1, Table 1). The initial assessment at our specialist unit ranged from 0.75 to 39 years of age. Only three patients were seen in childhood, and the remaining after puberty; four of these patients had previously undergone bilateral gonadectomy. The patient with normal‐appearing female external genitalia was referred at 21 years of age due to primary amenorrhea (patient 8, Table 1).

**Table 1:** Clinical Phenotype of Ten 46,XY DSD Patients and One 46,XX Testicular DSD Patient in Whom *NR5A1* Mutations Were Identified

Patient	Karyotype	Age at first evaluation (years)	External genitalia	Mullerian structures	Age at surgery	Sex of rearing	Family
1	46,XY	0.5	Atypical Phallus 3 cm, perineal hypospadias, palpable testes	A	Genitoplasty at 1.6 years	Male	NA
2^a^	46,XY	0.7	Atypical Clitoromegaly, palpable gonads	A	Gonadectomy at 9 m^a^; Genitoplasty at 2 years	Female	NA
3	46,XY	4.9	Atypical Single perineal opening, palpable gonads	A	Gonadectomy and Genitoplasty at 4.9 years	Female	M – WT, F – NA
4^a^	46,XY	12	Atypical Clitoromegaly (2.0 cm),single perineal opening, palpable gonads	A	Gonadectomy at 3 years	Female	NA
5	46,XY	12.3	Atypical Clitoromegaly (4.0 cm), two perineal openings, no palpable gonads. Virilization at puberty	A	Gonadectomy and Genitoplasty at 12.5 years	Female	Mother is a carrier of the mutation and presented with POI
6	46,XY	13	Atypical Clitoromegaly (3cm), labial fusion, no palpable gonads	P	Gonadectomy and Genitoplasty at 15.5 years	Female	Mother is a carrier of the mutation, F – NA
7^a^	46,XY	16.7	Atypical Clitoromegaly, left gonad palpable	P	Gonadectomy at 14 years; Genitoplasty at 15 years	Female	NA
8	46,XY	21	Female genitalia	P	Not performed	Female	NA
9^a^	46,XY	26	Atypical Phallus 6.5 cm, perineal hypospadias, palpable testes	A	Genitoplasty at 26 years	Female to Male (9 years)	NA
10^a^	46,XY	39	Atypical, clitoromegaly (3cm), two perineal opening	A	Gonadectomy at 14 years; Genitoplasty at 39 years	Female	NA
11^a^	46,XX	59	Atypical, no palpable gonads	A	Genitoplasty in childhood	Male	NA

a Surgery performed previously to the evaluation in our center.

P, present; A, absent; F, father; M, mother; NA, not available; years, years of age; POI, primary ovarian insufficiency.

Basal hormonal evaluation was available for seven patients. Gonadotropin levels were available in three prepubertal and in four postpubertal patients. All postpubertal patients had elevated gonadotropin levels, mainly FSH. Among four postpubertal patients, basal testosterone levels were normal for age in three (360, 155, and 414 ng/dl, respectively) and low in the single patient with normal female external genitalia (patient 8). A stimulation test with hCG was performed in four patients (two prepubertal and two pubertal) and in all of them testosterone levels increased adequately (Table 2).

**Table 2:** Hormonal Profiles of Brazilian 46,XY DSD and 46,XX Testicular DSD Patients in Whom *NR5A1* Mutations Were Identified

Patient	Age (years)	FSH (U/l)	LH (U/l)	Testosterone (ng/dl)	
				Basal	After hCG
1	0.5	NA	NA	11	458
	13	6	5	NA	508
2^a^	0.7	2	4	NA	12
3	4.9	5	<0.6	14	169
4^a^	12	NA	NA	NA	NA
5	12.3	77	13	414	NA
6^a^	13	NA	NA	NA	277
7	13	69	24	155	295
8	21	60	16	14	NA
11^a^	26	21	14	360	499
9^a^	39	NA	NA	NA	NA
10^a^	59	45	28	178	NA

Chronological age corresponding to the hormonal evaluation.

Conversion factors to SI units: T, ng/dl to nmol/l, multiply by 0.0347

a Surgery performed previously to the evaluation in our Center

NA, not available.

Seven patients underwent bilateral gonadectomy; age at gonadectomy ranged from 9 months to 15.5 years. The two patients who were raised as males had inguinal testes and underwent bilateral orchiopexy. Gonadectomy was not performed in one patient who discontinued follow‐up care. In three patients, a uterus was identified by pelvic ultrasound or MRI (Table 1).

None of the patients had symptoms or signs of adrenal insufficiency, and normal basal adrenal function was documented in six patients.

A family history of DSD was presented in three cases. Patient 6 had two paternal cousins with atypical genitalia. Patient 7 reported two second‐degree cousins with primary amenorrhea. Patient 5's mother, who also carried the *NR5A1* variant, developed ovarian insufficiency at 39 years.

### Clinical and Hormonal Characteristics of the 46,XX DSD Patient Bearing a Pathogenic *NR5A1* Variant

Although mutations in *NR5A1* have been reported in a small proportion of women with primary ovarian insufficiency (POI), *NR5A1* mutations were not identified in 70 patients with POI in our cohort. However, the previously described p.Arg92Trp NR5A1 mutation was found in heterozygous state in one 46,XX patient with testicular DSD. This patient (Patient 11, Table 1) was first seen in our Unit when he was 59 years old. He was born with atypical genitalia and had undergone masculinizing genitoplasty during childhood. Spontaneous secondary sexual characteristics such as phallic enlargement were absent and testosterone replacement was started at 15 years of age. Bilateral mastectomy was performed at 18 years of age. He did not have symptoms or signs of adrenal insufficiency, and basal adrenal profiling was normal. Peripheral blood leukocyte DNA was PCR‐analyzed for Y‐specific genes such as *SRY, TSPY, AMGY*, *DYZ3*, *DYS280*, and *DYS1*, all of which were not amplified and therefore considered absent. Pelvic MRI performed at 59 years of age revealed small bilateral testes in the inguinal regions; a uterus was not identified.

### Molecular Characteristics of Novel NR5A1 Defects

**Figure 1:**
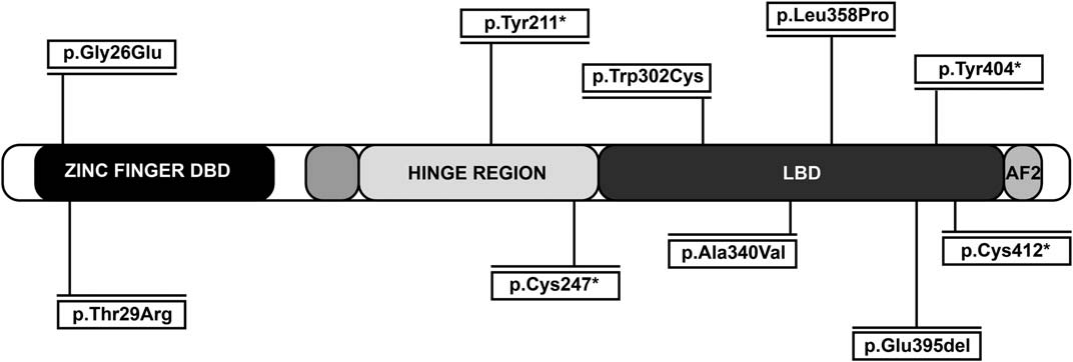
The ten novel mutations identified in 46,XY DSD patients and their localization on the NR5A1 protein. DBD, DNA‐binding domain; LBD, ligand‐binding domain; AF2, activation function domain.

Ten novel heterozygous *NR5A1* mutations were identified in 46,XY DSD patients (Fig. 1), including five nonsynonymous variants (p.Gly26Glu, p.Thr29Arg, p.Trp302Cys, p.Ala340Val, p.Leu358Pro), four stop‐gain variants (p.Tyr211*, p.Cys247*, p.Tyr404*, p.Cys412*), and one frameshift variant (p.Glu395del) (Table 3, Fig. 1). In three cases DNA samples were obtained from patients’ mothers; two of them carried *NR5A1* mutations (Table 3).

**Table 3:** Molecular Characterization of *NR5A1* Defects Identified in Heterozygous State in Ten 46,XY and in One 46,XX Testicular DSD Patients

Patient	Mutation (DNA)	Mutation (protein)	Domain	Functional impact	Bind energy
**8**	c.77G>A	p.Gly26Glu	DBD (1st zinc finger)	In silico prediction: damaging	**↓**
**9**	c.86C>G	p.Thr29Arg	DBD	In silico prediction:damaging	NA
**5**	c.663C>G	p.Tyr211*	Hinge	Truncated protein	NA
**2**	c.741C>A	p.Cys247*	Hinge	Truncated protein	NA
**7**	c.906G>C +c.437G>C	p.Trp302Cys +p.Gly146Ala	LBD Hinge	In silico prediction: damaging	**↑**
**1**	c. 1019C>T	p.Ala340Val	LBD	In silico prediction: damaging	NA
**3**	c.1073T>C	p.Leu358Pro	LBD	Impaired transactivation^fs a^	**↑**
**6**	c.1183_1185del	p.Glu395del	LBD	In silico prediction: damaging	**↓**
**10**	c.1212C>G +c.437G>C	p.Tyr404* +p.Gly146Ala	LBD Hinge	Truncated protein	**↑**
**11**	c.1236C>A	p.Cys412*	LBD	Truncated protein	NA
**4**	c.274C>T	p.Arg92Trp	DBD (A box)	Ref	NA

The alignment of the rows is according to mutation location in the NR5A1protein. Binding energy to the “ligand di‐pamitoyl‐3‐SN‐phosphatidylethanolamine of the mutated complex compared to the non‐mutated complex.

Functional studies of mutated NR5A1 activity shown a reduction in the transcriptional activity of Cyp11a1 and Cyp19 promoters.

↓, reduced bind energy; ↑, increased bind energy; DBD, DNA‐binding domain; LBD, ligand‐binding domain; NA, not available.

The previously described common nonsynonymous p.Gly146Ala variant (rs1110061) was identified in two patients in association with p.Trp302Cys and p.Tyr404* *NR5A1* mutations, respectively.

### In Silico Studies of the NR5A1 Variants Identified in 46,XY Patients

**Figure 2:**
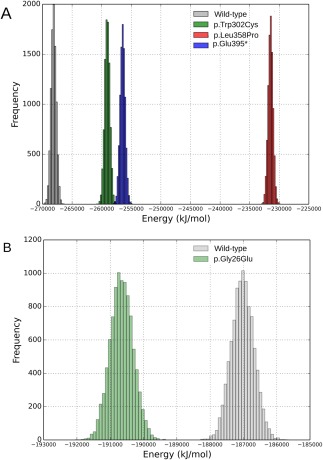
(**A**) Energy distributions of the native NR5A1 LBD domain and mutated molecules. A higher energy is associated with a less stable molecule compared to the native due loss of amino acid contacts. (**B**) Energy distributions of the native NR5A1 DBD domain and its p.Gly26Glu mutated molecule. A smaller energy is associated to a more stable molecule, compared to the native due additional amino acids contacts.

Primary sequence analyses showed that all NR5A1 mutated amino acids are extremely conserved among non‐redundant homologous and relative sequences. Regarding potential and binding energies studies, native and mutated energy distributions of the p.Gly26Glu, p.Trp302Cys, p.Leu358Pro, p.Tyr404*, and p.Glu395del variants were compared. The LBD domain mutations p.Trp302Cys and p.Tyr404* both led to a higher energy state and therefore to a less stable molecule in comparison with the wild‐type protein (Fig. 2).

The p.Gly26Glu variant, located in the DBD, establishes a polar contact not found in the original molecule, increasing its affinity for DNA binding and making the NR5A1 dissociation from the DNA helix difficult, thus potentially impairing transcriptional regulation. Interestingly, the p.Gly26Glu variant seems to stabilize the DBD, leading to a smaller energy compared to that of the native domain (Fig. 2). As a consequence, an opposite behavior was observed for binding energies. While all LBD mutated molecules studied increased the bind energy to the ligand di‐pamitoyl‐3‐SN‐phosphatidylethanolamine, p.Gly26Glu provided smaller binding energy to the DNA helix. Potential and binding energy means, variations, and overall conformations between mutated and native molecules are shown in Table 4.

**Table 4:** Variation of the Potential and Binding Energies Means Overall 9.000 Conformations Between Mutated and Native Molecules

**Mutation/domain**	**Δ*E* (kJ/mol)**	**Δ*E*_**bind**_ (kJ/mol)**
p.Gly26Glu/DBD	3633	−1746
p.Trp302Cys/LBD	9053	36
p.Leu358Pro/LBD	36,639	36
p.Glu395 del/LBD	11,656	59

All samples distributions passed Shapiro‐Wilk normality test, being means compared with Welsh's t‐test.

All resultant *p* values were less than 2.2 x 10^−16^.

**Figure 3:**
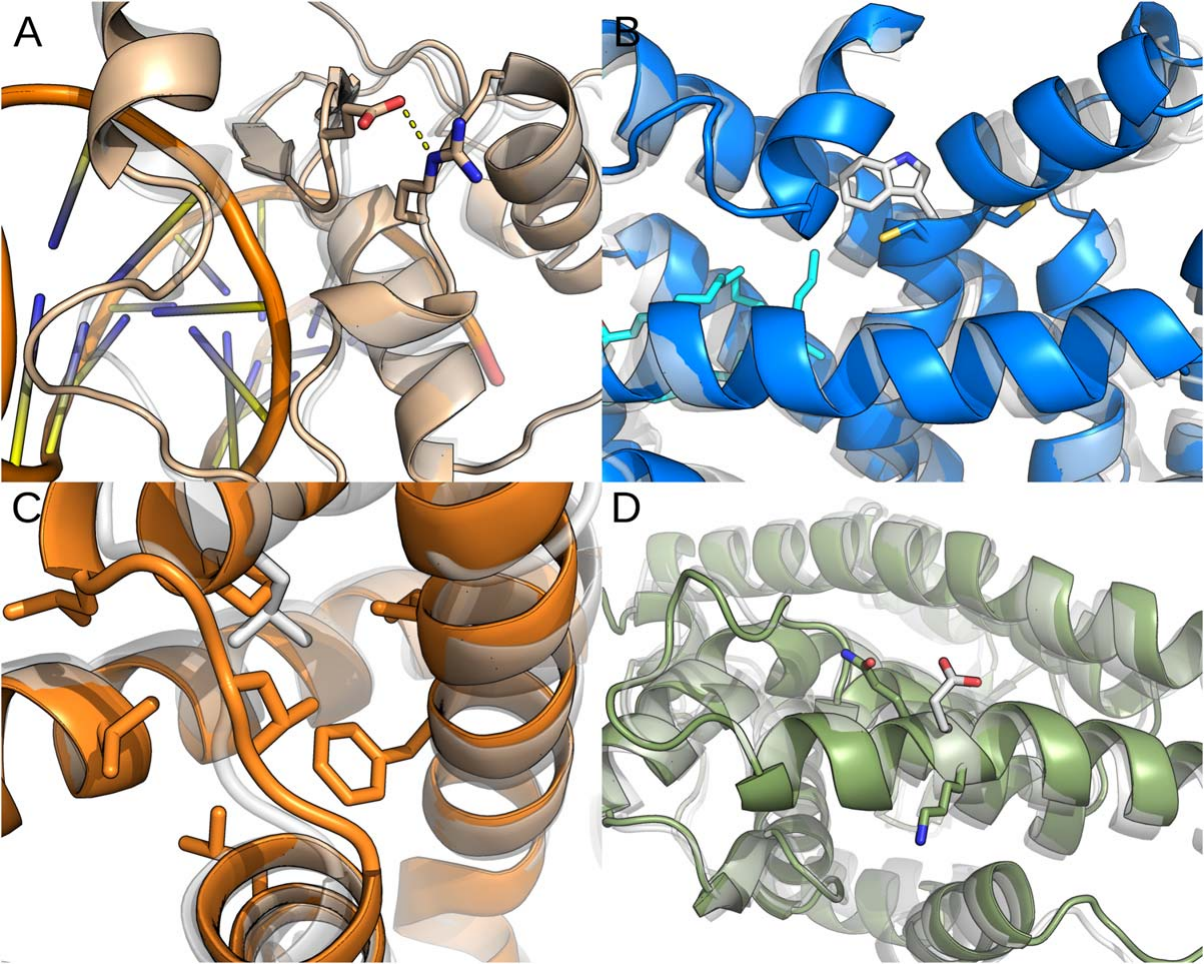
Superimposed Models: Superimposition of the native (white) and mutated smallest energy NR5A1 models (colored): (**A**) DBD domain showing Glu26 and Arg79 salt bridge; (**B**) LBD domain showing Try302Cys; (**C**) LBD hydrophobic network with Leu358Pro in the center; and (**D**) LBD helix‐9 rotated and unrolled half a turn with the Glu385 deletion.

Figure 3 presents the lowest energy molecular models of all mutated domains superposed to their respective native versions. Replacement of Gly by Glu at position 26 creates an additional contact not present in the original DBD domain. The negatively charged Glu26 Oε2 forms a salting bridge with the Arg79 Nε, which makes the β‐hairpin 21‐20 less flexible and closer to the ligand DNA. In the native LBD domain, the Try302imidazolic ring contributes to form a hydrophobic environment of the active site bottom cleft required to accommodate fatty ligands. Removal of such a bulky amino acid at that position induces a whole structural reorganization so that the central cavity of the protein shrinks, decreasing the occluded volume from 885 Å3 to 724 Å3. In turn, the p.Leu358Pro mutation is located in a hydrophobic core surrounded by four α‐helices. In the original molecule, Leu358 establishes several contacts with a network of hydrophobic residues. Although proline is a non‐polar amino acid, its side chain is too small compared to leucine and cannot effectively reach the surrounded hydrophobic residues. Therefore, many hydrophobic contacts were lost, which may explain the potential energy increasing. This change may be responsible for the reduction of transcriptional activity observed in the functional studies described below.

The variants p.Tyr211* and p.Cys247* are located in hinge region of NR5A1 that is important for stabilizing the LBD and interaction with other proteins that control NR5A1 transcriptional activity.

Finally, the Glu385 deletion is located within LDB helix‐9, which is in the opposite direction of the ligand cleft. Together with the N‐terminal helix‐1, these two external helices tightly embrace the entire domain. In addition, the removal of the Glu385 slightly rotates the helix‐9 axial axis; weakening several lateral contacts and making the helix unroll half a turn. All these modifications are predicted to be transmitted throughout the whole domain decreasing its affinity to the ligand.

The results of the molecular simulation analyses strongly support the hypothesis that all these novel mutations potentially interfere with the activity of the NR5A1 protein. Its structural stability and its ability to bind the di‐palmitoyl‐3‐SN‐phosphatidylethanolamine and probably other fat can be affected by the presence of the mutations. The function of such fat acid has not been cleared; however, it certainly provides additional contacts and stability to the whole molecule to perform its activities. Altogether, the simulation results strengthen the hypothesis that the studied mutations are very likely deleterious.

### In Vitro Functional Studies of the p.Leu358Pro NR5A1 Variant

**Figure 4:**
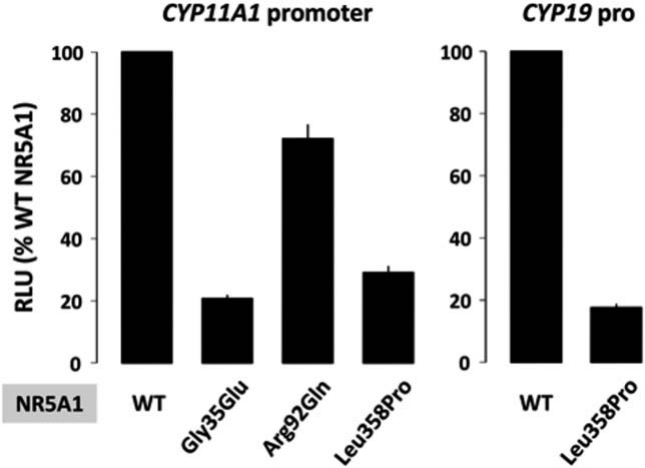
Transcriptional activation of target gene promoters by wild‐type (WT) and mutant NR5A1 in human embryonic kidney TSA‐201 cells. In vitro activation of *CYP11A1* and *CYP19* gene promoters by the novel NR5A1 mutant p.Leu358Pro was reduced, in comparison to WT NR5A1 (29% and 18% of WT, respectively). Notably, the degree of transcriptional activity impairment at the *CYP11A1* promoter by p.Leu358Pro NR5A1 was comparable to that of the well‐known NR5A1 mutant p.Gly35Glu (Lin et al., 2007) and more severe than p.Arg92Gln (Achermann et al., 2002). Results represent the mean (SEM) for three assays, each performed in triplicate, and shown relative to wild‐type transactivation. RLU, relative luciferase units.

In vitro studies showed a markedly impaired transcriptional activity of the p.Leu358Pro mutant NR5A1, with a reduction of more than 70% and 80% in the transactivation of the *Cyp11a1* and *Cyp19* promoters, respectively (Fig. 4).

## Discussion

### *NR5A1* Mutations in 46,XY Individuals

Since the first 46,XY DSD patient with the p.Gly35Glu *NR5A1* mutation described by Achermann et al. (1999), the spectrum of phenotypes associated with *NR5A1* mutations has greatly expanded. This initial patient bearing the heterozygous p.Gly35Glu mutation presented with adrenal failure and gonadal dysgenesis with persistent Mullerian derivatives (Woo et al., 2015), a phenotype that closely resembled the phenotype observed in murine *Nr5a1* knockout models (Luo et al., 1994). Gonadal dysgenesis and adrenal insufficiency were also present in the second reported NR5A1‐related 46,XY DSD, in a patient bearing the homozygous p.Arg92Gln mutation (Achermann et al., 2002).

The expected phenotype associated with *NR5A1* mutations was shifted when a heterozygous 8‐bp microdeletion was found in a 46,XY DSD patient who presented with clitoromegaly, absence of uterus and gonads but normal adrenal function (Correa et al., 2004). After this first report, the study of several cohorts of individuals with 46,XY DSD has shown that adrenal insufficiency is a rare finding in patients with NR5A1 defects (Lin et al., 2006; Guran et al., 2016).

Reported heterozygous *NR5A1* mutations support the model that partial NR5A1 dysfunction can result in several degrees of impaired Leydig cell function and androgen biosynthesis, leading to predominantly abnormal gonadal phenotypes, which can range from complete testicular dysgenesis with Mullerian structures, through mild clitoromegaly or atypical genitalia without Mullerian derivatives, to proximal hypospadias associated with undescended testis (Köhler et al., 2009), or even micropenis with absent gonads (Philibert et al., 2007).

Currently*, NR5A1* mutations represent one of the most frequent defects associated with 46,XY gonadal dysgenesis, accounting for up to 20% of cases (Suntharalingham et al., 2015).

More than 80 different *NR5A1* variants, distributed across the full length of the protein, have been described and the majority are nonsynonymous mutations (Pedace et al., 2014; Tantawy et al., 2014; Woo et al., 2015; Fabbri et al., 2016). Most of these mutations are located in the DBD and are in a heterozygous state or compound heterozygous state with the p.Gly146Ala (rs1110061) variant, with the exception of two mild mutations described in homozygous state (Achermann et al., 2002; Soardi et al., 2010). These findings reinforce the concept that NR5A1 dosage is critical to normal gonadal development. However, a clear correlation between the location of a mutation, its in vitro functional performance, and the associated phenotype is not observed. Indeed, family members bearing the same *NR5A1* mutation may present with variable phenotypes (Warman et al., 2011).

There are some hypotheses to explain the phenotypic variation associated with a similar *NR5A1* mutation. First, interallelic association with the known p.Gly146Ala polymorphism (rs1110061) may further reduce NR5A1 activity and contribute to more severe phenotypes (WuQiang et al., 2003; Hasegawa et al., 2004; Wada et al., 2006; Reuter et al., 2007; Köhler et al., 2008; Lourenço et al., 2009; Bashamboo et al., 2010a; Paris et al., 2011; Camats et al., 2012). However, two patients in our cohort also bore the p.Gly146Ala variant besides the pathogenic NR5A1 variants, p.Trp302Cys and p.Tyr404*, and their phenotype was not strikingly distinct or more severe.

The contribution of other genetic modifiers has also been suggested to explain phenotypic variability. Exome sequencing analyses of DSD patients have identified pathogenic variants or variants of uncertain significance in several genes involved in sexual development (Bashamboo et al., 2010b; Bashamboo et al., 2014). In a 46,XY patient with atypical external genitalia, palpable inguinal gonads, absent uterus in pelvic ultrasonography, and poor testosterone response to hCG stimulation, Mazen and colleagues (2016) identified, by exome sequencing, the previously described p.Arg313Cys *NR5A1* mutation in compound heterozygous state with a p.Gln237Arg *MAP3K1* variant. This *NR5A1* mutation was previously reported in association with mild hypospadias (Allali et al., 2011), and a possible digenic inheritance was proposed to explain the phenotypic heterogeneity (Mazen et al., 2016).

Progressive androgen production and virilization in adolescence has been observed in several XY patients with *NR5A1* mutations, in contrast to the severe undervirilized external genitalia found in most patients (Cools et al., 2012; Gabriel Ribeiro de Andrade et al., 2014; Tantawy et al., 2014; Fabbri et al., 2016). The almost normal testosterone levels after hCG stimulation or at pubertal age suggest that NR5A1 action may be less implicated in pubertal steroidogenesis than during fetal life. NR5A1 is expressed in the both fetal and adult Leydig cells, but differences in its actions at both stages are not completely known. The study of a hypomorphic mouse model of *Nr5a1* indicated differential impairment of fetal and adult Leydig cell development, whereby *Nr5a1* may regulate the differentiation of fetal Leydig cells, whereas in the adult it may regulate progenitor cell formation and/or survival (Karpova et al., 2015). Different actions of NR5A1 in fetal and postnatal Leydig cells populations might contribute to the switch from birth to pubertal phenotypes.

In contrast, fetal Sertoli cell function seems to be preserved in the most patients with heterozygous *NR5A1* mutations, based on the common observation of absent Müllerian derivatives and primitive seminiferous tubules on histology. In our cohort, three of 10 patients had Müllerian derivatives although in one of them the uterine volume remained infantile after three years of estrogen replacement.

The reviewed data of seventy‐two 46,XY DSD patients with *NR5A1* mutations reported in the literature, for whom information on presence or absence of Müllerian derivatives was available, suggested that Müllerian derivatives are present in about 24% of the cases (Pedace et al., 2014; Tantawy et al., 2014; Woo et al., 2015; Fabbri et al., 2016). However, persistently elevated FSH levels after puberty found in all patients studied suggest an impairment of Sertoli cells function in post pubertal age (Pedace et al., 2014).

Male infertility has been also related to the presence of *NR5A1* defects (Bashamboo et al., 2010a; Ferlin et al., 2015). Patients with moderate/severe oligospermia or azoospermia and *NR5A1* mutations may have normal testosterone and normal low or low inhibin B levels, but they are at potential risk of deterioration of testicular hormonal secretion with age and may need counseling regarding preservation of sperm and regular monitoring of endocrine function (Bashamboo et al., 2010a).

The presence of ovotestis was described in a 46,XY girl, with the 9q33.3‐q34.1 deletion encompassing *NR5A1* and *LMX1B* genes causing genitopatellar syndrome associated with female external genitalia and clitoromegaly (Schlaubitz et al., 2007).

### *NR5A1* Mutations in 46,XX Subjects

The first reported 46,XX patient with a *NR5A1* mutation (p.Arg255Leu) presented with isolated adrenal insufficiency, but a follow‐up description of ovarian function at postpubertal age is lacking (Biason‐Lauber and Schoenle, 2000). Subsequently, it was shown that *NR5A1* mutations are also a genetic cause of POI, with phenotypes ranging from primary to secondary amenorrhea, associated with infertility, hypoestrogenism, and elevated gonadotropin levels (Lourenço et al., 2009; Camats et al., 2012) Sisters and mothers of 46,XY DSD patients carrying heterozygous *NR5A1* mutations may also develop premature ovarian failure (Lourenço et al., 2009; Camats et al., 2012; Fabbri et al., 2016).

Several genes required for ovarian steroidogenesis, and follicle growth and maturation are regulated by *NR5A1*, explaining why mutant *NR5A1* may lead to progressive loss of ovarian function and female reproductive capacity (Lourenço et al., 2009; Camats et al., 2012; Fabbri et al., 2016). Women bearing *NR5A1* defects should receive appropriate counseling and fertility guidance, enabling potential oocyte cryopreservation, due to the risk of developing ovarian insufficiency later in life.

In contrast to its prominent pathogenic role in 46,XY DSD, NR5A1 defects are a rare cause of sporadic POI of unknown etiology in women (1.4–1.6%). (Suntharalingham et al., 2015) Sixteen different *NR5A1* variants distributed across the full length of the protein have been described in patients with premature ovarian failure, and the majority of them are nonsynonymous mutations (Suntharalingham et al., 2015)

The spectrum of disorders associated with *NR5A1* variants in 46,XX individuals was further expanded by the demonstration of the specific recurrent heterozygous p.Arg92Trp NR5A1 variant, in association with variable degrees of testis development in 46,XX patients from unrelated families (Baetens et al., 2016; Bashamboo et al., 2016; Igarashi et al., 2017). The p.Arg92Trp *NR5A1* mutation was described in ten *SRY* negative 46,XX DSD patients, six with testicular DSD and four with ovotesticular DSD. This mutation was found in two familial cases: the first one comprising two 46,XX ovotesticular DSD sisters and the second one involving one 46,XX testicular DSD patient and his 46,XY sister with partial gonadal dysgenesis. In these two families a maternal inheritance was identified. Atypical genitalia was the most frequent presentation (six patients), but a phenotypic spectrum ranging from female external genitalia with clitoromegaly (one patient), to male external genitalia with micropenis (two patients) or penoscrotal hypospadias (one patient) was observed. Three of eight patients had a uterus, and two had a hemiuterus. Gonads were palpable in seven patients and the other three 46,XX ovotesticular patients had bilateral abdominal ovotestis.

The p.Arg92Gln NR5A1 mutation, located at the same residue, was previously described in homozygous state in a 46,XY phenotypic female with adrenal insufficiency and in homozygous state in a 46,XX girl with early‐onset primary adrenal insufficiency. This variant was also present, in heterozygous state, in her phenotypically normal sister, mother, and father. (Achermann et al., 2002; Guran et al., 2016) Differences in the transactivating activity of the p.Arg92Gln and the p.Arg92Trp mutants may be responsible for the significant phenotypic differences observed (Igarashi et al., 2017) Nevertheless, the contribution of modifying allelic variants in other genes involved in gonadal development may not be completely excluded.

In vitro assays demonstrated that the p.Arg92Trp mutant lost its binding capacity to a consensus NR5A1 response element and reduced activation of some minimal promoters (*Amh, Cyp11a1*) (Bashamboo et al., 2016). This mutated protein was also less sensitive to NR0B1‐induced suppression on the *SOX9* TESCO element, leading to testis formation (Igarashi et al., 2017). These findings suggest a dysregulation of normal female gonadal development by p.Arg92Trp NR5A1, tipping the balance toward testis development in 46,XX individuals.

## Conclusion

In a single‐center Brazilian cohort, we have identified 10 novel *NR5A1* mutations in 46,XY DSD patients and the previously described p.Arg92Trp mutation in one 46,XX testicular DSD patient. Additionally, no mutations were found in 70 patients with POI. Our findings reinforce those of previous reports and contribute to expanding our understanding of NR5A1‐related phenotypes in humans.

*NR5A1* mutations are associated with a wide spectrum of disorders of gonadal development, ranging from DSD to oligo/azoospermia in 46,XY individuals and 46,XX ovotesticular and testicular phenotypes to primary ovarian failure in 46,XX individuals. The most frequent phenotype in 46,XY patients is atypical or female external genitalia with clitoromegaly, palpable gonads, and absence of Müllerian derivatives. Postnatally, Leydig cells function seems to be preserved in several patients with *NR5A1* mutation, while Sertoli and germ cells are more profoundly affected. The severely undervirilized external genitalia frequently seen at birth, contrasts with spontaneous virilization at puberty in several patients. Phenotypic variability observed in patients with similar NR5A1 defects may be related to the association with genetic modifiers or pathogenic variants in other testis/ovarian‐determining genes.

## Supporting information

Supporting Information
