# Supplementary material for: Wide spectrum of NR5A1‐related phenotypes in 46,XY and 46,XX individuals
Source: Birth Defects Res C Embryo Today. 2016 Dec 29;108(4):309–20. doi: 10.1002/bdrc.21145 (PMC5347970; doi:10.1002/bdrc.21145)
Supplement: Supplementary file 1 — Supporting Information [file BDRC-108-309-s001.doc]

**Supplemental data**

Genomic DNA was extracted from peripheral blood leukocytes by the proteinase K-SDS salting-out method (24).

**Sanger sequencing**

The entire NR5A1 encoding region (NM_004959/ ENST00000373588) and the exon-intron boundary areas were PCR-amplified using specific primers. The PCR products were purified with “ExoSAP-IT” enzyme (GE Healthcare Life Sciences, Buckinghamshire, UK) and sequenced using standard dideoxy chain termination methods with ABI Prism BigDye Terminator Cycle Sequencing Ready Reaction Kit 3.1 (Life Technologies, Foster City, CA, USA). Control DNA samples were obtained from 100 fertile males.

**Target massively parallel sequencing (TMPS)**

A panel of 61 candidate genes was selected, including 39 genes already associated with human DSD, 8 involved in gonadal determination pathways, 3 associated with DSD in mice and 11 genes involved in ovarian differentiation. Their entire coding regions, and respective 50-bp boundaries, were captured using custom Sure Select Target Enrichment System Kit (Agilent) (Gnirke et al., 2009). Sequencing was performed in the Illumina MiSeq platform. Paired-end reads (2 x 300) were aligned to the hg19 assembly of the human genome with BWA-MEM (Li and Durbin 2009). Aligned reads were sorted and converted to the BAM format using the bamsort tool from the biobambam2 suite (https://launchpad.net/biobambam2). Mean coverage was over 95 x for all the samples and more than 96% of the RefSeq gene coding regions were covered at 20 x or deeper. Single-nucleotide variants and small INDELs were simultaneously called in all samples using Freebayes (https://github.com/ekg/freebayes). Annotation of the variants was performed with ANNOVAR (Wang et al., 2010).

In prioritizing the most likely pathogenic variants, we filtered out those with a minor allele frequency > 0.5% in publicly available populational databases ExAC (Song et al., 2016) and 1000 Genomes (Auton et al., 2015). We then focused on protein-altering exonic and putative splice site variants. Accordingly the American College of Medical Genetics and Genomics guidelines, we classified variants into five main categories: pathogenic, likely pathogenic, variants of uncertain clinical significance (VUS), likely benign, and benign (Richards et al., 2015). To assess the possible impact on protein structure and function of the novel non synonymous variants, we used 8 in silico algorithms (SIFT (Ng and Henikoff 2006), PolyPhen2 (Adzhubei et al., 2010), Mutation Taster (Schwarz et al., 2010), Mutation Acessor , FATHMM (Shihab et al., 2013), LRT (Chun and Fay 2009), LR score, radial SVM (Dong et al., 2015)), 3 conservation scores (GERP++ (Davydov et al., 2010), SiPhy (Garber et al., 2009) and PhyloP (Siepel et al., 2006)) and one Ensemble score (CADD) (Kircher et al., 2014).

**Functional Studies of p.Leu358Pro allelic variant**

**Transient gene expression assays**

Expression vectors containing the p.Leu358Pro mutants were generated by site-directed mutagenesis (QuikChange, Stratagene, Amsterdam, The Netherlands) using wild-type (WT) pCMXSF-1 as a template. The entire coding sequence was confirmed by direct sequencing prior to functional analyses. Transient transfection studies were performed in 96-well plates using lipofectamine 2000 (Invitrogen) and a dual-luciferase reporter assay system (Promega). Studies were performed in tsa201 human embryonic kidney cells by transfecting empty, WT, or mutant NR5A1 expression vectors (2 ng/well; p.Gly35E, p.R92Q, p.Leu358Pro) with the SF-1-responsive minimal promoter of Cyp11a and Cyp19 linked to luciferase (100 ng/well). Cells were lysed 24 h following transfection and assayed for luciferase activity (Dual Luciferase Reporter Assay system, Promega; FLUOstar Optima, BMG Labtech, Aylesbury, UK), with standardization for Renilla co-expression. Results are shown as the mean of three independent experiments, each performed in triplicate.
